# 

**Published:** 1887-01

**Authors:** 


### CONTRIBUTORS TO VOL. VIII.

Harrison, Allen, M. D.

Richard W. Burke, A. V. D.

Henry C. Chapman, M. D.

A. W. Clement, V. S.

W. A. Conklin, Ph.D.

Cooper Curtice, D. V. M.

J. T. Duncan, M. D.

L. Webster Fox, M. D.

Mark L. Frey, V. S.

Alexander Glass, M. D.

William S. Gottheil, M. D.

A. S. Heath, M. D.

R. S. Huidekoper, M. D.

Daniel D. Lee, M. D. V.

Joseph Leidy, M. D., LL.D.

A. H. Logan, V. S.

R. Lovell, V. S.

J. H. Miller, V. S.

T. Wesley Mills, M. D., L.R.,C.P.

C. Pittfield Mitchell, M. D.

H. W. Mitchell, M. D.

C. M. O'Leary, M. D., LL. D.

J. O'Leary, M. D.

William Osler, M. D.

Johann W. Schutz, M. D.

E. O. Shakespeare, M. D.

R. W. Shufeldt, M. D., U. S. A.

Theobald Smith, M. D.

Edward C. Spitzka, M. D.

J. Bland Sutton, M. D.

N. S. Townsend, M. D.

Thomas Walley, M. R. C. V. S.

W. L. Zuill, M. D., D. V. S.

### INDEX.

Abortion, iron weed as a cause of .......... 95
Abscess tubercula in the vertebræ .......... 276
Actinomycosis in man .......... 294
Allen Harrison, anatomy of the elephant .......... 153
American health association .......... 90
    veterinary college .......... 196
Anasarca .......... 122
Aneurism of the aorta .......... 79
    — mesenteric artery .......... 230
Animals, medicine as practiced by .......... 96
Anthrax poison, earth worms as carriers of .......... 294
Aorta, rupture of the .......... 273
Army, veterinary service of .......... 138
Artery coronary, rupture of .......... 187
    mesenteric, rupture of .......... 186
Autopsies at Central Park menagerie .......... 71, 183, 267

Baboon chacma, autopsy of .......... 267
Bacteriology .......... 293
    practical methods in .......... 241
Baird, S. F.—obituary .......... 395
Barrett, Michael—obituary .......... 287
Barsati or atrophic carcinoma .......... 60, 164
Bear, autopsy of .......... 268
Beri-beri .......... 80
Birds, muscles used in classification of .......... 321
Books and pamphlets received .......... 100, 205, 291, 403
Brachial artery, embolism of the .......... 274
Breeding and feeding .......... 47
Burke, R. W.—aneurism of the mesenteric artery .......... 230
    barsati or atrophic carcinoma .......... 60, 164
    kamri or paraplegia .......... 367
    surra or progressive pernicious anæmia .......... 223

Camel, hydatids in a .......... 266

Carbunculus in horses .......... 363

Case department .......... 71, 183, 267, 385

Cat, whooping cough in .......... [illegible]93

Cattle, cerebro-spinal meningitis in.......... 73
growers association.......... 68
Chapman, H. C.—observations on the brain of the elephant.......... 149
Chauveau, Jean, B. A.—biography.......... 174
Chicago veterinary college.......... 283
Clement, A. W.—autopsies.......... 186
notes in some of the centres of veterinary education..... 144
Colon of the horse.......... 349
Conventions in Chicago.......... 66
Cow as a source of scarlet fever.......... 293
Curtice Cooper—hydatids in a camel.......... 266
Cysts sebaceous.......... 11

Dentigerous growths..........382, 387
Diseases of birds beaks.......... 181
Dogs, dislocation of the eye-ball in .......... 246
vaccination and distemper in.......... 276

Editorial Department..........66, 176, 262, 374
Elasticity as a conservative force.......... 132
Elephant, anatomy of.......... 153
brain of.......... 149
lungs and digestive apparatus of.......... 157
Endocarditis chronica.......... 79
Equine spinal meningitis.......... 387

Fracture femur.......... 77
lumbar vertebræ.......... 77
radius in brood mare.......... 268
Frey, Mark L.—spinal meningitis in horses .......... 387
Fox, L. Webster,—comparative ophthalmology.......... 43

Glanders, etiology of.......... 81
Glass, Alexander.—lanoline.......... 158
Gotthiel, William S.—autopsies..........183, 267
on some tænia of the quadrumana.......... 136

Hæmatoza, recent research on .......... 176
Hæmoglobinuria from cold.......... 82
Heath, A. S., breeding and feeding.......... 47
Horses, carbunculus in.......... 363
cause of influenza in.......... 248
colon of.......... 349
disease at Coonong.......... 72
gangrene in.......... 273
gastric calculus in.......... 279
hæmoglobinuria in.......... 82
poisoning of, by Indian pea.......... 78
Horns, cutaneous.......... 11

Huidekoper, R. S.—anasarca........ 122
Fracture of radius in mare........ 268
lungs and digestive apparatus of elephant........ 156
scalma........ 344
Hyæna, autopsy of........ 71
Hydatids in camel........ 266

Impregnation........ 275
Influenza........ 270
pectoralis, the contagium of........ 188
International Medical Congress........ 179

Kamri, or paraplegia........ 367

Lanolin........ 158
Lee, Daniel D.—carbunculus in horses........ 363
dislocation of the eye-ball in dogs........ 246
umbilical and ventral entero-epiplocele........ 161
Leidy, Joseph.—tape-worms in birds........ 1
Logan, A. H.—dentigerous growths........ 382
Lovell, R.—influenza catarrhal fever........ 270

Mechanical horse shoeing........ 98
Medicine as practiced by animals........ 96
Meyers, Charles A—obituary........ 394
Microbe of rabbit septicæmia........ 24
Miller, J. H.—nasal polypus........ 387
Mills, T. Wesley—elasticity as a conservator force in the animal organism, 132
snake poison from a chemico-physiological point of view, 38
Missouri association........86, 195
Monkey macaque, autopsy........ 184
toque, autopsy........ 183
weeping capuchin, autopsy........185, 18[illegible]
Montreal veterinary college........ 198
medical association........194, 199

Nasal polypus........ 386
National cattle growers' association........ 68
veterinary and sanitary association........66, 84
Nervous lactation........ 75
New Jersey State veterinary society........ 390
New York college of veterinary surgeons........ 197
Notes on the anatomy of the Indian elephant........ 149

Obituary........ 287
Ocelot, autopsy of........ 267
Œsophagus, diseases of........ 103
Ohio State veterinary medical society........ 392
Omental hernia........ 78

Ontario veterinary medical association.................................. 92
college.....................................91, 280
society.................................... 89
Open hock joint.................................................. 77
Ophthalolmogy, comparative.......................................... 43
Osler, William—recent researches on hœmatozoa........................ 176
scarlatina............................................. 262

Paradoxure, autopsy of............................................ 267
Pelvic lipoma...................................................... 76
melanosis.................................................. 76
Penis, double...................................................... 76
Pennsylvania State veterinary medical association....... .......... 190, 393
Pharmaceuticals for veterinary practice.............................. 98
Pleuro-pneumonia, the contagium of................................ 189
Pneumonia lobular................................................... 76
Proceedings veterinary medical societies......................84, 190, 280, 390
Progress of veterinary science.................................95, 293

Recent researches on scarlatina.................................... 262
Report of the English commission on Pasteur......................... 374
Reviews.....................................................99, 200, 288, 396
Royal (Dick) veterinary college...................................... 97

Salmon, D. E.—biography........................................... 257
Sarcomatous diathesis............................................... 77
Scalma............................................................ 344
Scarlet fever, the cow as a source of................................ 293
Schutz, Johann W.—cause of influenza pectoralis in horses... ......... 248
Scottish metropolitan veterinary medical society..................... 93
Sebaceous glands, physiology of...................................... 272
Selections from foreign periodicals .............................78, 188, 271
Shakespeare, E. O.—some familiar and practical methods in bacteriology, 241
Sheep, changes produced in the lungs of.............................. 271
Shufeldt, R. W.—a review of the muscles used in the classification of birds......................................... 321
comparison of a series of skulls of wild and domesticated turkeys................................ 207
diseases of birds' beaks.................................. 181
the veterinary service of the U. S. army.................. 138
Smith, Theobald—contribution to the study of the microbe of rabbit septicæmia........................................................ 24
Snake poison........................................................ 38
Spinal meningitis in horses......................................... 387
Spitzka, E. C.—report of the English commission on Pasteur............ 374
Statistics of hospital and out patient service.....................74, 387
Strain cervicle ligaments............................................ 76
Surra or progressive pernicious anæmia ............................. 223

Sutton, J. B.—comparative study of sebaceous cysts and cutaneous horns, 11
teratomata an ætiological study ........ 295

Tæniæ of the quadrumana........ 186
Tape worms in birds........ 1
Technics of animal vaccination........ 233
Teratomata, an ætiological study........ 295
Thrombosis of the iliac arteries........ 79
Townsend, N. S.—lung worms in sheep........ 56
Turberculosis in the grouse........ 180
Turkeys, comparison of skulls of wild and domesticated ........ 207

United States veterinary medical association........71, 192
Umbilical and ventral entero-epiplocele........ 161

Veterinary college, American ........ 196
Montreal........ 198
New York........ 197
Ontario........ 91
Royal (Dick)........ 97
department Harvard University........ 285
University Pennsylvania........74, 263, 285
education, notes on........ 144
national and sanitary association........66, 84
medical association, Missouri........86, 195
Montreal........194, 199
New Jersey........ 87
Ontario........ 92
Pennsylvania........ 190
United States........71, 192
society, Ontario........ 89
societies proceedings........84, 190, 280, 390
Scottish........ 93
quackery........ 81
society meetings........ 180

Walley, Thomas—colon of the horse, its diseases and derangements........ 349
organic diseases of the œsophagus........ 103
Whooping cough in a cat........ 293
Worms, tape........ 1

Zuill, W. L.—technics of animal vaccination........ 233

### BOOKS AND PAMPHLETS RECEIVED.

Contagious and Parasitic Diseases of Animals. Published by the State Board of Health, Maine.

Preventable disease circulars, including typhoid and scarlet fever, diphtheria and small-pox. They will be furnished by applying to A. G. Young, M. D.. Secretary, Augusta, Maine.

Professor Walley's Address at the Opening of the 63d Winter Session, Royal Veterinary College, Edinburgh.

Constitution and By-Laws of the Missouri State Association of Veterinary Science and Comparative Medicine.

Studies from the Biological Laboratory, John Hopkins University, edited by H. Newell Martin, M.D., and W. K. Brooks, Ph. D. Vol. III., No. 8.

Transactions of the American Dermatological Association, 1886.

Is Electrolysis a Failure in the Treatment of Urethial Structures, by Robert Newman, M.D., New York.

Cases in Orthopædic Surgery, by Ap. Morgan Vance, M.D., Louisville.

LARYNGOLOGY AND ITS COGNATE BRANCHES IN AMERICA, AND THE SIMPLEST AND MOST EFFICIENT TREATMENT OF DIPTHERIA, by W. H. Daly, M.D., Pittsburg.

OSTEOLOGICAL NOTE UPON THE YOUNG OF GEOCOCCYX CALIFORNIANUS AND A SKULL OF A NAVAJO CHILD, by R. W. Shufeldt, M.D., U. S. A.

By Prof. B. G. Wilder, NOTES ON THE BRAIN, THE COLLOCATION OF A SUTURE AND FISSURE IN THE HUMAN FŒTUS.

HUMAN CEREBRAL FISSURES, THEIR RELATIONS AND NAMES AND THE METHOD OF STUDYING THEM.

THE PAROCCIPITAL FISSURE,

By Prof. T. Wesley Mills, INNERVATION OF THE HEART OF THE SLIDER TERRAPIN, *Pseudemys Rugosa.*

THE RHYTHM AND INNERVATION OF THE HEART OF THE SEA TURTLE.

THE HEART OF THE FISH COMPARED WITH THAT OF MENOBRANCHUS WITH SPECIAL REFERENCE TO REFLEX INHIBITION AND INDEPENDENT CARDIAC RHYTHM.

THE PHYSICIANS' VISITING LIST, for 1887, Philadelphia, 1886, P. Blakiston, Son & Co.

